# Association between Markers of Synovial Inflammation, Matrix Turnover and Symptoms in Knee Osteoarthritis: A Cross-Sectional Study

**DOI:** 10.3390/cells10071826

**Published:** 2021-07-20

**Authors:** Xiaotian Yang, Christian S. Thudium, Anne-Christine Bay-Jensen, Morten A. Karsdal, James van Santen, Nigel K. Arden, Thomas A. Perry, Stefan Kluzek

**Affiliations:** 1 Department of Rehabilitation Medicine, Sir Run Run Shaw Hospital, Zhejiang University School of Medicine, Hangzhou 310016, China; 2 Botnar Research Centre, Nuffield Department of Orthopaedics, Rheumatology and Musculoskeletal Sciences, University of Oxford, Oxford OX3 7LD, UK; james.vansanten@ndorms.ox.ac.uk (J.v.S.); nigel.arden@ndorms.ox.ac.uk (N.K.A.); thomas.perry@ndorms.ox.ac.uk (T.A.P.); stefan.kluzek@nottingham.ac.uk (S.K.); 3 Immunoscience, Nordic Bioscience, DK-2730 Herlev, Denmark; cst@nordicbio.com (C.S.T.); acbj@nordicbio.com (A.-C.B.-J.); mk@nordicbio.com (M.A.K.); 4 Centre for Sport, Exercise and Osteoarthritis Research Versus Arthritis, Nuffield Department of Orthopaedics, Rheumatology and Musculoskeletal Sciences, University of Oxford, Oxford OX3 7LD, UK; 5 MRC Lifecourse Epidemiology Unit, University of Southampton, Southampton General Hospital, Southampton SO17 1BJ, UK; 6 Division of Rheumatology, Orthopaedics and Dermatology, School of Medicine, University of Nottingham, Nottingham NG7 2UH, UK

**Keywords:** osteoarthritis, biomarkers, synovitis, symptoms

## Abstract

To investigate the association between markers of synovial inflammation and matrix turnover (MRI-based and serum biomarkers) and knee symptoms in established knee osteoarthritis (KOA). This cross-sectional study utilised data from a randomised, multicentre placebo-controlled trial (UK-VIDEO) of vitamin D therapy in symptomatic KOA. Data on serum biomarkers, type III collagen degradation (C3M), metabolite of C-reactive protein (CRPM) and cartilage oligomeric matrix protein (COMP), were available at baseline whilst contrast-enhanced (CE) MRI data were acquired in a subsample at baseline and annually. Knee symptoms were assessed using WOMAC at all visits. We examined the cross-sectional association between knee symptoms and three MRI-based and three serum markers of synovitis and matrix turnover, respectively. A total of 447 participants were included in the serum and 136 participants in the MRI analyses. MRI-defined medial perimeniscal synovitis was positively associated with knee pain and, suprapatellar and medial perimeniscal synovitis with knee function in multivariate analysis. We observed a statistically significant, negative association between a higher concentration of serum C3M and CRPM and knee pain, respectively. Furthermore, the highest CRPM quartile was negatively associated with knee function. Our findings suggest that, in established painful radiographic KOA, MRI-defined medial perimeniscal and suprapatellar synovitis were positively associated with knee symptoms. Serum-based C3M and CRPM markers were negatively associated with knee symptoms. Pain fluctuations are common in KOA and a better understanding of the relationship between markers of synovitis and matrix turnover and knee symptoms would facilitate a more accurate assessment of temporal changes in disease progression.

## 1. Introduction

Osteoarthritis (OA), the most common form of arthritis, is a leading cause of disability in adult populations [1]. OA is a heterogeneous joint disease characterised by fluctuating knee pain and a reduction in physical function [2,3]. OA presents as cartilage degeneration, remodelling of the subchondral bone and localised inflammation [4]. The emerging evidence suggests, in the most part, that synovitis and fibrosis plays an important role in the pathogenesis of OA and is related to clinical symptoms and structural progression [5,6,7,8,9,10,11].

Knee pain has been shown to be one of the strongest predictors of radiographic progression in radiographic knee OA (KOA) [12]. More so, evidence from both observational and interventional studies suggest that synovial inflammation measured on MRI contributes to knee pain [13,14,15]. Synovial fibrosis associated with collagen synthesis and matrix turnover contributes heavily to joint pain [10]. Currently, the gold standard for defining synovitis is histological assessment of samples obtained through biopsy, though this approach is limited. To accurately assess the structure–pain relationship, there is a great need to identify less invasive markers, preferably imaged-based and/or serum biomarkers, that can be used as surrogates of synovial inflammation and matrix turnover.

Biomarkers can fulfil different purposes as defined by the BIPEDS classification: burden of disease, investigational, prognostic, efficacy of intervention, diagnostic and/or safety biomarkers [16].

MRI is commonly performed to assess synovitis severity. Contrast-enhanced (CE) MRI allows for the assessment of synovial thickening and enhancement after administration of intravenous contrast agent, as distinct from effusion, and has been shown in MRI-histological studies to correlate with synovitis in people with KOA [17,18].

Measures of whole joint synovitis and synovitis at specific regions have been shown to correlate with knee pain [19,20]. There is some evidence suggesting that synovitis of the infrapatellar fat pad and suprapatellar region have stronger relationships with knee pain in symptomatic KOA compared to whole-knee joint synovitis possibly suggesting more localised inflammatory changes [21,22]; a non-linear association has been observed between MRI synovitis score and incidence of radiographic KOA [23]. This concept is not new and there is further data to suggest more local synovial inflammatory changes occur with structural damage; for instance, chronic synovial inflammation adjacent to injured meniscal tissue has been linked with knee pain [24]. Large MRI studies confirmed that medial perimeniscal synovial hypertrophy is a common feature of painful medial KOA [25]. The most recent review of observational studies showed a correlation between histologically defined synovial inflammation and MRI assessed synovitis with histological severity correlating with MRI-based whole knee semi-quantitative scoring in a population with established KOA [26]. The CE-MRI scores correlated best with inflammatory infiltrates of synovial tissue [26]. Although associated with higher haematoxylin and eosin (H&E)-based histological grading, changes in MRI-based whole knee semi-quantitative scoring may not necessarily represent changes in inflammatory characteristics associated with inflammatory phenotyping; there is a possibility of OA pathology and patient heterogeneity [27].

In addition to imaging, other collagen synthesis and matrix turnover-related markers identified in serum, specifically degradation fragments of type III collagen degradation (C3M), metabolites of C-reactive protein (CRPM) and cartilage oligomeric matrix protein (COMP), have the potential to serve as sensitive measures linked to knee pain. There is evidence to suggest that levels of C3M and CRPM are elevated in people with OA compared to healthy controls, though this relationship may be independent of radiographic OA severity [28]. Furthermore, the concentration of C3M has been shown to be lower in participants with severe radiographic KOA compared with participants with less severe radiographic structural changes [28], and similar decreased levels have been shown in patients with moderate to severe synovitis [29].

Comparably, high serum COMP values were associated with increased risk of incident KOA independently of age and BMI [30] and synovitis has been shown to have the strongest effect on COMP levels in established KOA [31]. High COMP and CRPM concentrations have been associated with the progression of radiographic knee and hip OA, respectively [32]. Concentrations of CRPM and C3M have been shown to be higher in participant with OA compared with non-OA controls [28]. Loeser et al. reported positive associations for change in C3M and CRPM with change in pain and function in overweight and obese individuals with KOA who lost weight due to diet and/or exercise [33]. COMP, CRPM and C3M have some potential to act as diagnostic and prognostic markers and to act as a measure of efficacy of intervention in KOA. However, it is still unclear whether these biomarkers are associated with differences in pain and function (burden of disease) in people with symptomatic KOA. We hypothesized that markers of synovitis and matrix turnover are associated with knee symptoms in established symptomatic KOA.

Therefore, our aim was to investigate the association between MRI-based and serum-based markers of synovial inflammation and matrix turnover and symptoms in participants with established KOA.

## 2. Materials and Methods

### 2.1. Study Population

We used data from the Vitamin D Evaluation in Osteoarthritis (UK-VIDEO) trial. The VIDEO protocol was approved by the Scotland A Research Ethics Committee and the trial was registered with EudraCT: ref. 2004-000169-37, ISRCTN94818153, CTA No. 11287/0001/001. A total of 474 participants aged between 50 and 79 years with symptomatic KOA, defined as pain in the knee for most days in the last month, were recruited across five clinical sites. Details of the original study including inclusion and exclusion criteria and study design, as well as the main trial outcomes, have been previously published [34]. In this secondary analysis using cross-sectional data, participants with available biochemical markers (C3M, CRPM and COMP) and CE-MRI of index knee as well as WOMAC pain and function scores were included.

### 2.2. MRI Acquisition and Assessment

MRI acquisition protocols and imaging scoring systems used for the assessment of synovial thickness in the current study have been reported previously [19]. In brief, the extent of synovial inflammation was measured on the axial and/or sagittal TI-weighted (T1-W) fat suppressed (FS) CE-MRI in the index knee. Synovial enhancement was scored semi-quantitatively (grade 0 to 3) at 11 different sites across the knee, including Hoffa’s (infrapatellar) fat pad, parapatellar and supra-patellar regions, on the sagittal and/or axial planes [35]. The scores were categorized as follows: 0 = normal synovium, 1 = mild synovitis (<2 mm thickness), 2 = moderate synovitis (2–4 mm thickness), and 3 = severe synovitis (>4 mm thickness). Of the 136 participants included in the CE-MRI subgroup analysis, a single visit was assessed per participant. Semi-quantitative CE-MRI readings of synovitis were examined at the baseline visit for 74 participants (54.41%), at the 12-month visit for 35 participants (25.74%), at the 24-month visit for 23 participants (16.91%), at the 30-month visit for two participants (1.47%) and at the 36-month visit for two participants (1.47%).

### 2.3. Biochemical Markers

Blood samples were collected at baseline only. All biochemical markers were measured in serum by Nordic Bioscience, Herlev, Denmark. Enzyme-linked immunosorbent assay (ELISA) applying neoepitope-specific monoclonal antibodies was used to quantify C3M and CRPM (Nordic Bioscience, Herlev, Denmark). Sera were obtained from venous fasting blood during baseline visit and stored at −80 °C. Serum concentrations of C3M and CRPM were measured blindly by manual competitive ELISAs, developed by Nordic Bioscience. Briefly, for C3M, which measures the neo-epitope of MMP degraded type III collagen fragment KNGETGPQGP [28], streptavidin pre-coated 96-well plates (Roche Diagnostics, Mannheim, Germany) were coated with 0.4 ng/mL biotin labelled peptide (biotin-KNGETGPQGP) and incubated for 30 min at 20 °C. After washing, the standard, controls and serum samples (diluted 1:4 in incubation buffer) were added, followed by peroxidase-conjugated antibody. The mixture of sample–antibody was incubated at 20 °C for 60 min. The sample–antibody mix was removed from the wells and peroxidase reaction was visualized by 3,3′,5,5′-tetramethylbenzidine (TMB, Kem-En-tec, Taestrup, Denmark) at 20 °C and stopped with sulfuric acid after 15 min and measured. Calibration curves were plotted using a four-parametric mathematical fit model.

The CRPM assay measures the MMP generated neo-epitope KAFVFPKESD of C-reactive protein [36]. The CRPM ELISA followed the same procedure as C3M; however, a different peroxidase-conjugated antibody (NB94-1A7) and coater (KAFVFPKESDK-biotin) was used. Serum COMP was measured using AnaMar Medical ELISA kit (Uppsala, Sweden). This is a one-step sandwich ELISA (Human COMP^®^ ELISA) to detect COMP. The intra- and inter-assay variations were below 4% and 8%, respectively. The quantification range was determined internally by the lab as 2 (lower limit of quantification) to 35 (upper limit of quantification) U/L. All samples were measured in duplicate and within the measuring range when diluted between 1 in 4 and 1 in 10. Samples were rerun if intra assay coefficients of variability (CVs) were above 15% or if their values were outside the quantification range. Each plate included two kit controls, as well as three internally identified quality control samples. From these, the inter-assay CVs were monitored and controlled for being <10%.

All samples were measured in duplicate and were ensured to maintain the proper concentration when diluted 10 times and allowing coefficients of variation (CV) < 15% and were otherwise re-measured. Inter- and intra-assays CV were below 10% and 14%, respectively, for C3M and below 9% and 10%, respectively, for CRPM.

### 2.4. Statistical Analysis

Descriptive statistics were used to assess the clinical, imaging and serum data. Normally distributed variables were described using means and standard deviations (SD) and non-normally distributed variables described using median and inter-quartile ranges (IQR). Standard linear regression analyses adjusted for potential confounders (age, BMI) were conducted to assess the associations between markers of synovitis (CE-MRI synovitis site-specific scores) and matrix turnover (C3M, CRPM and COMP) and pain (WOMAC pain subscale) and physical function (WOMAC function subscale), respectively. We formally tested the assumptions of linear regression to assess the suitability of our models and there were no violations. Participants with grade 2 and 3 MRI synovial thickness scores, for each respective joint region, were combined due to the small sample size of each respective category. Biochemical markers (C3M, CRPM and COMP) were categorised into quartiles due to the suspected non-linear effects on the outcomes. We did not adjust for the allocation of treatment intervention as there was no evidence to support an effect of vitamin D supplementation on structural modification [37].

All statistical analyses were performed using STATA/IC package version 15.1 (StataCorp., College Station, TX, USA).

## 3. Results

### 3.1. Participants Characteristics

**Figure 1:**
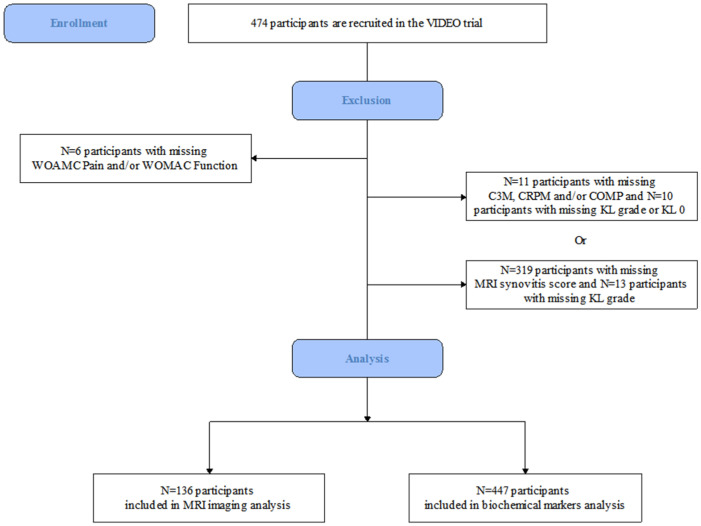
Flow chart of the study. Western Ontario and McMaster Universities Osteoarthritis Index (WOMAC), type III collagen degradation (C3M), metabolite of C-reactive protein (CRPM) and cartilage oligomeric matrix protein (COMP), Kellgren–Lawrence (KL).

Of 474 participants recruited to VIDEO, 447 (94.30%) had complete serum biomarker data, were of the Kellgren–Lawrence (KL) grades 1 to 4 and had knee pain and function data at baseline. A total of 136 (28.69%) participants had additional CE-MRI data across visits; there were no significant differences between these groups (Figure 1).

The demographic and clinical characteristics of the included participants, by MRI and serum analyses, are shown in Table 1. Over 60% were female and had a median BMI of over 28 kg/m^2^. Whilst the inclusion criteria for the original VIDEO trial specified that study participants must have a KL grade of 2–3, upon re-evaluation of the x-rays during the original study, some study participants were graded as KL 1 and 4.

### 3.2. MRI-Defined Semi-Quantitative Synovitis Thickness

Semi-quantitative measures of CE-MRI defined synovial thickness in three regions of the knee were found to be associated with knee pain and/or knee function, respectively (Table 2 and Table 3).

Compared to those with no evidence of synovitis on CE-MRI, we observed a statistically significant positive association between CE-MRI defined moderate-to-severe medial perimeniscal synovitis and knee pain (9.83, 95% CI 1.33 to 18.32) after adjustment for age and BMI, with an increase in synovitis severity associated with worsening knee pain (Table 2). We observed no statistically significant association between suprapatellar and infrapatellar synovial thickness and knee pain, respectively.

In a fully adjusted model, CE-MRI defined mild suprapatellar synovial thickness score was positively associated with knee function (10.07, 95% CI 0.15 to 20.00), with increasing synovial thickness score associated with worsening knee function, when compared with absent suprapatellar synovitis (Table 3). No statistically significant association was observed between CE-MRI defined infrapatellar and medial perimeniscal synovitis and knee function.

### 3.3. Biochemical Markers

We observed statistically significant, negative associations between the concentration of two serum biomarkers of matrix turnover (C3M and CRPM) and knee symptoms (Table 4 and Table 5).

Statistically significant negative associations were observed between the third quartile of C3M (−6.27, 95% CI −11.11 to −1.44) and CRPM (−7.34, 95% CI −12.18 to −2.49) biomarkers and knee pain when compared with the lowest quartile, respectively (see Table 4). After adjusting for age and BMI, associations between the third C3M quartile and the third CRPM quartile and knee pain remained statistically significant (−6.97, 95% CI −11.67 to −2.26; −7.4, 95% CI −12.12 to −2.68) when compared with the lowest quartile, respectively. In addition, we observed a negative statistically significant association between the second C3M quartile and the lowest quartile of C3M (−5.17, 95% CI −9.87 to −0.47) after adjusting for age and BMI.

A statistically significant negative association was observed between the third CRPM quartile and knee function in univariate (−6.29, 95% CI −11.62 to −0.95) and adjusted models (−5.94, 95% CI −10.98 to −0.90), respectively, compared to the lowest CRPM quartile (Table 5). There was no evidence of a statistically significant association between C3M and knee function.

Lastly, there was no association between COMP and knee pain and function, respectively (Table 4 and Table 5).

## 4. Discussion

In this cross-sectional study, markers linked with knee joint inflammation were associated with knee symptoms in symptomatic KOA. We observed a positive association between semi-quantitatively assessed medial perimeniscal synovitis, measured on CE-MRI, and knee pain, with increasing severity of synovial thickness associated with worse knee pain. Furthermore, CE-MRI-defined suprapatellar synovitis was positively associated with knee function. Using whole-body serum biomarkers of matrix turnover, we observed a negative statistically significant association between knee pain and serum C3M and CRPM biomarkers, respectively. Lastly, we observed a negative, statistically significant association between the third quartile of the CRPM and knee function when compared to the lowest CRPM quartile.

Previous data suggests that medial synovitis, assessed on CE-MRI, is associated with knee pain [19] though there has been little work to identify which medial sites are likely to be the main contributors to knee pain. Our data agrees with the findings reported by Wallace et al. [19], which utilised a separate sample of the VIDEO study. Our study, however, goes beyond describing the association between the severity of site-specific medial perimeniscal synovitis and knee symptoms exclusively. Previous studies indicate that medial perimeniscal synovitis is associated with meniscal damage of the posterior horns [38] and severe medial chondropathy [25]. These data suggest that the association between pain and medial perimeniscal synovitis may be attributed to a synovial response to local chondral damage. Further research is required to distinguish whether the association is influenced by medial structural damage including injury to the medial menisci or excessive loading through the medial compartment of the knee. Unfortunately, soft tissue injuries were not assessed as part of the primary VIDEO protocol and so we could not adjust for this in our analyses.

In contrast to the findings of previous studies [21,22,39], there was no evidence of an association between knee pain and suprapatellar and infrapatellar synovitis in our study. In one previous study [39], volume of the infrapatellar fat pad was measured by CE-MRI. Here, the authors report that synovitis volume of the infrapatellar fat pad showed stronger correlations with knee pain than semi-quantitatively scored synovitis [39]. Kaukinen et al. [21] and Wang et al. [22] used semi-quantitative scoring to grade effusion-synovitis measured by non-CE MRI. In the current study, synovitis was measured by CE-MRI, which could explain the conflicting findings. To more precisely characterize the role of synovitis, quantitative assessment of synovial tissue volume may be a promising method. Furthermore, quantitative measures have been reported to be more sensitive in describing the association with knee symptoms in symptomatic KOA [20].

The different methodology might also explain why our results related to function contrast with the findings of the previous study [39]. In the current study, no statistically significant association was observed between infrapatellar and medial perimeniscal synovitis and knee function, though we did observe a relationship at other sites, i.e., the suprapatellar site. A possible explanation for this could be due, in part, to the method used to assess synovitis severity. The method outlined by Guermazi et al. [40] requires a single maximal thickness measurement be taken along the axis of synovitis within Hoffa’s region which may not truly reflect the severity of synovitis for a structure that is highly dynamic.

In our study, an increase in C3M and CRPM was associated with less severe knee pain compared to participants with the lowest quartile of the serum biomarkers. The study participants within the first quartile of the serum biomarkers had high levels of knee pain with more limited function when compared with other quartiles. A previous study showed that the concentration of C3M and CRPM were elevated in people with OA when compared to healthy controls [28]; however, in those with KOA, no association between change in C3M and CRPM and, change in pain was found over 18 months [33]. Furthermore, in a previous study, none of those serologic markers correlated with the intensity or duration of knee pain, nor radiological grade [41]. High CRPM has been previously associated with the degree of centralized sensitization after adjustment for age, sex, BMI and high-sensitivity C-reactive protein, but not with clinical pain intensity in established OA [41]. Additionally, high blood levels of CRPM have been reported to be predictive of contra-lateral knee OA [42].

A possible explanation is that the joint’s symptomatology is associated with a balance between persistent local inflammation and a higher level of collagen synthesis and matrix turnover associated with fibrosis. The biomarkers of fibrosis, in classical fibrotic disease like lung fibrosis and liver fibrosis, are the same as in our study [43]. Therefore, a positive association between synovitis and pain is reasonable; a negative association between higher markers associated with fibrosis and matrix turnover is also supported by this hypothesis. Another potential hypothesis is that fibroblasts in chronic synovial inflammation switch from production of type III to type I collagen. This would result in increased release of type III collagen and CRP neoepitopes in early remodelling and a drop of concentration of those neoepitopes with increased volume of established synovial inflammation. Furthermore, type III collagen may also originate from cartilage, suggesting that the reduced cartilage volume in late-stage patients is translating into a smaller pool of type III collagen fragments [44].

A statistically significant association was observed between CRPM and WOMAC function with an increase in concentration of CRPM associated with an improvement in knee function. We observed (see Appendix A, Appendix B and Appendix C) higher concentrations of CRPM in KL grades 1–2, which could in part explain the statistically significant negative association. A previous study of overweight and obese participants with KOA who lost weight from diet and/or exercise showed that a change in C3M and CRPM was positively associated with an improvement in WOMAC function [33], with a decrease in serum concentration associated with worsening symptoms. One potential reason for our conflicting findings could be due to the inclusion in our study of participants with KL grades 1 rather than exclusively including participants with grades 2–3. To address this, we performed a sensitivity analysis which, after omitting KL grades 1 (n = 118), showed that the negative statistically significant association between CRPM and function remained.

Although CE-MRI was acquired which allowed for accurate differentiation of inflamed synovium from joint effusion, our study had several potential limitations. Firstly, the number of subjects who took part in the MRI analysis was small; subsequently, the reported confidence intervals are wide. Not all subjects recruited across the five participating sites had MRIs acquired with CE-MRIs acquired at the Southampton site only. Furthermore, soft tissue injuries were not assessed (e.g., meniscal tears, bone marrow lesions (BMLs)) and so we were unable to account for injury/trauma in our models; knee pain and related symptoms may derive from such structures. In addition, our study design was cross-sectional meaning that we were unable to determine a causal relationship between the respective synovial surrogates and knee symptoms. Further study is required to confirm these findings and an analysis of change vs. change (i.e., change in synovial surrogates vs. knee symptoms) would be beneficial. We were unable to perform such an analysis as serum biomarkers were only collected at baseline. A further limitation was that we did not assess the presence of OA at other joint sites. X-rays and clinical diagnoses for other joint sites were not assessed. It was not part of the VIDEO protocol to assess OA at other sites beyond the knee. The reported relationships between blood biomarkers and disease of a specific joint are complicated by the fact that participants might have disease at other sites.

## 5. Conclusions

Our study showed that in established symptomatic KOA, CE-MRI-assessed medial perimeniscal and suprapatellar synovitis were positively associated with knee symptoms. Lower concentrations of serum-based C3M and CRPM markers were associated with higher pain. Further work is, however, needed to confirm the relationship between the serum concentration of markers of synovial inflammation and matrix turnover and knee symptoms in symptomatic KOA.

## Figures and Tables

**Table 1:** Characteristics of study participants.

Characteristic	MRI Imaging (n = 136)	Serum Biomarkers (n = 447)
Age (years) ^1^, median (IQR)	65 (60–70)	64 (58–69)
Sex, n (%)		
Male	51 (34.23)	177 (39.60)
Female	98 (65.77)	270 (60.40)
BMI (kg/m^2^), median (IQR)	28.32 (26.44–31.97)	28.73 (25.86–32.30)
WOMAC pain score ^2^, median (IQR)	28.3 (16.50–45.37)	29.80 (17.00–44.40)
WOMAC function score, median (IQR)	33.94 (17.18–53.29)	32.47 (18.94–50.56)
Kellgren–Lawrence grade, n (%)		
1	25 (18.38)	118 (26.40)
2	57 (41.91)	172 (38.48)
3	37 (27.21)	131 (29.31)
4	17 (12.50)	26 (5.82)
Total synovitis score (0–33) ^3^, median (IQR)	12 (7–16)	13 (6–16)
C3M (ng/mL) ^4^, median (IQR)	31.67 (26.47–39.03)	34.09 (28.36–41.22)
CRPM (ng/mL) ^4^, median (IQR)	7.70 (6.56–10.66)	9.35 (7.47–11.75)
COMP (ng/mL) ^4^, median (IQR)	12.01 (10.08–14.03)	11.50 (9.54–13.59)

^1^ Values are presented as medians (interquartile range) for non-normally distributed variables, and counts (percentage) for categorical variables. Body mass index (BMI), interquartile range (IQR), Western Ontario and McMaster Universities Osteoarthritis Index (WOMAC), type III collagen degradation (C3M), metabolite of C-reactive protein (CRPM) and cartilage oligomeric matrix protein (COMP). ^2^ WOMAC: visual analogue scale (VAS) was used to score knee symptoms from 0 to 100 units (0 = no pain/disability, 100 = high pain/disability). ^3^ n = 74 participants in the serum biomarkers dataset; higher scores denote more severe synovitis. ^4^ n = 74 participants in the MRI imaging dataset.

**Table 2:** Association between site-specific synovial thickness score and knee pain.

		Crude				Adjusted ^1^			
	Group	Coefficient	*p*	95% Conf Interval		Coefficient	*p*	95% Conf Interval	
Suprapatellar	Mild (n = 35)	1.371	0.783	−8.454	11.197	1.447	0.767	−8.187	11.082
	Moderate and Severe (n = 71)	0.713	0.870	−7.886	9.313	1.288	0.764	−7.193	9.77
Infrapatellar	Mild (n = 46)	4.963	0.211	−2.849	12.776	4.003	0.311	−3.777	11.782
	Moderate and Severe (n = 34)	1.244	0.774	−7.292	9.780	1.224	0.774	−7.184	9.631
Medial perimeniscal	Mild (n = 54)	3.860	0.343	−4.158	11.877	5.217	0.192	−2.653	13.086
	Moderate and Severe (n = 40)	8.330	0.058	−0.280	16.939	9.825	0.024 *	1.325	18.324

^1^ Adjusted for age and body mass index (BMI). Higher scores denote more severe knee pain. * *p* < 0.05.

**Table 3:** Association between site-specific synovial thickness score and knee function.

Crude						Adjusted ^1^			
	Group	Coefficient	*p*	95% Conf Interval		Coefficient	*p*	95% Conf Interval	
Suprapatellar	Mild (n = 35)	9.926	0.068	−0.74	20.592	10.072	0.047 *	0.146	19.999
	Moderate and Severe (n = 71)	4.012	0.397	−5.323	13.347	4.653	0.294	−4.086	13.391
Infrapatellar	Mild (n = 46)	7.919	0.068	−0.586	16.424	5.758	0.161	−2.327	13.843
	Moderate and Severe (n = 34)	−0.556	0.906	−9.848	8.737	−0.851	0.847	−9.589	7.887
Medial perimeniscal	Mild (n = 54)	4.776	0.289	−4.095	13.648	7.207	0.087	−1.054	15.469
	Moderate and Severe (n = 40)	6.343	0.190	−3.184	15.869	8.320	0.067	−0.603	17.244

^1^ Adjusted for age and body mass index (BMI). Higher scores denote worse knee function. * *p* < 0.05.

**Table 4:** Association between biochemical markers of matrix turnover and knee pain.

Crude						Adjusted ^1^			
	Group	Coefficient	*p*	95% Conf Interval		Coefficient	*p*	95% Conf Interval	
C3M	Second quartile	−4.744	0.055	−9.580	0.092	−5.166	0.031 *	−9.866	−0.466
	Third quartile	−6.272	0.011 *	−11.108	−1.436	−6.966	0.004 *	−11.671	−2.261
	Fourth quartile	0.271	0.913	−4.576	5.118	−1.691	0.486	−6.452	3.070
CRPM	Second quartile	−4.138	0.094	−8.985	0.708	−3.352	0.163	−8.071	1.367
	Third quartile	−7.338	0.003 *	−12.184	−2.491	−7.403	0.002 *	−12.124	−2.682
	Fourth quartile	−3.277	0.186	−8.134	1.580	−3.967	0.100	−8.700	0.766
COMP	Second quartile	−1.183	0.635	−6.075	3.709	−1.818	0.453	−6.573	2.937
	Third quartile	−0.025	0.992	−4.918	4.867	−1.086	0.657	−5.881	3.709
	Fourth quartile	0.609	0.807	−4.295	5.512	−0.724	0.768	−5.539	4.091

^1^ Adjusted for age and body mass index (BMI). All comparisons are made against those of the lowest quartile of the specific serum markers. Type III collagen degradation (C3M), metabolite of C-reactive protein (CRPM) and cartilage oligomeric matrix protein (COMP). * *p* < 0.05.

**Table 5:** Association between biochemical markers of matrix turnover and knee function.

Crude						Adjusted ^1^			
	Group	Coefficient	*p*	95% Conf Interval		Coefficient	*p*	95% Conf Interval	
C3M	Second quartile	−1.266	0.643	−6.622	4.091	−1.773	0.490	−6.818	3.272
	Third quartile	−2.970	0.276	−8.326	2.387	−3.846	0.135	−8.896	1.205
	Fourth quartile	1.710	0.532	−3.658	7.079	−1.147	0.659	−6.258	3.964
CRPM	Second quartile	−5.246	0.054	−10.585	0.092	−3.786	0.141	−8.827	1.256
	Third quartile	−6.285	0.021 *	−11.623	−0.947	−5.941	0.021 *	−10.984	−0.898
	Fourth quartile	−3.464	0.204	−8.814	1.886	−4.088	0.113	−9.144	0.969
COMP	Second quartile	0.878	0.748	−4.488	6.244	−0.118	0.963	−5.175	4.938
	Third quartile	0.632	0.817	−4.734	5.998	−1.436	0.580	−6.535	3.664
	Fourth quartile	3.186	0.245	−2.193	8.564	0.713	0.784	−4.408	5.834

^1^ Adjusted for age and body mass index (BMI). All comparisons are made against those of the lowest quartile of the specific serum markers. Type III collagen degradation (C3M), metabolite of C-reactive protein (CRPM) and cartilage oligomeric matrix protein (COMP). * *p* < 0.05.

**Figure A1:**
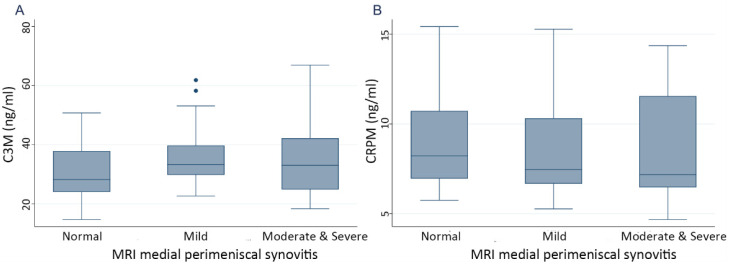
Biochemical marker level by grade of medial perimeniscal synovitis. (**A**) Type III collagen degradation (C3M); (**B**) metabolite of C-reactive protein (CRPM).

**Figure A2:**
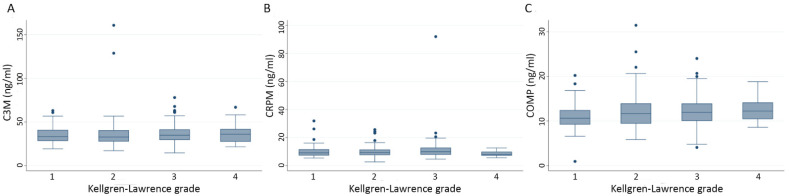
Biochemical marker level by Kellgren–Lawrence grade. (**A**) Type III collagen degradation (C3M); (**B**) metabolite of C-reactive protein (CRPM); (**C**) cartilage oligomeric matrix protein (COMP).

**Figure A3:**
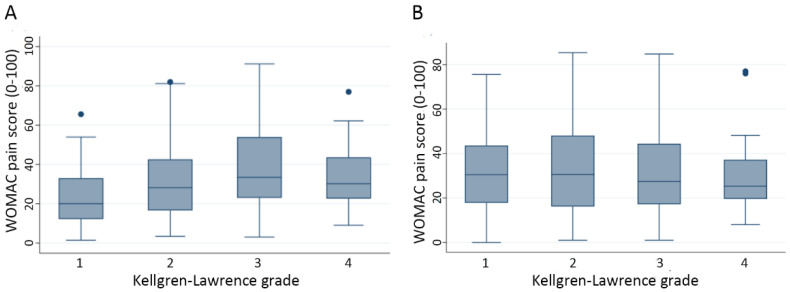
WOMAC pain score by Kellgren–Lawrence grade. (**A**) MRI imaging dataset; (**B**) biochemical markers dataset.

## Data Availability

The data generated during the current study are available from the corresponding author on reasonable request.

## References

[1] Neogi T. (2013). The epidemiology and impact of pain in osteoarthritis. Osteoarthr. Cartil..

[2] Felson D.T. (2006). Clinical practice. osteoarthritis of the knee. N. Engl. J. Med..

[3] Driban J.B., Sitler M.R., Barbe M.F., Balasubramanian E. (2010). Is osteoarthritis a heterogeneous disease that can be stratified into subsets?. Clin. Rheumatol..

[4] Lories R.J., Luyten F.P. (2011). The bone-cartilage unit in osteoarthritis. Nat. Rev. Rheumatol..

[5] Sellam J., Berenbaum F. (2010). The role of synovitis in pathophysiology and clinical symptoms of osteoarthritis. Nat. Rev. Rheumatol..

[6] Guermazi A., Hayashi D., Roemer F.W., Zhu Y., Niu J., Crema M.D., Javaid M.K., Marra M.D., Lynch J.A., El-Khoury G.Y. (2014). Synovitis in knee osteoarthritis assessed by contrast-enhanced magnetic resonance imaging (MRI) is associated with radiographic tibiofemoral osteoarthritis and MRI-detected widespread cartilage damage: The MOST study. J. Rheumatol..

[7] Roemer F.W., Guermazi A., Felson D.T., Niu J., Nevitt M.C., Crema M.D., Lynch J.A., Lewis C.E., Torner J., Zhang Y. (2011). Presence of MRI-detected joint effusion and synovitis increases the risk of cartilage loss in knees without osteoarthritis at 30-month follow-up: The MOST study. Ann. Rheum. Dis..

[8] Collins J.E., Losina E., Nevitt M.C., Roemer F.W., Guermazi A., Lynch J.A., Katz J.N., Kent Kwoh C., Kraus V.B., Hunter D.J. (2016). Semiquantitative imaging biomarkers of knee osteoarthritis progression: Data from the Foundation for the National Institutes of Health Osteoarthritis Biomarkers Consortium. Arthritis Rheumatol..

[9] Rim Y.A., Ju J.H. (2020). The role of fibrosis in osteoarthritis progression. Life.

[10] Remst D.F., Blaney Davidson E.N., van der Kraan P.M. (2015). Unravelling osteoarthritis-related synovial fibrosis: A step closer to solving joint stiffness. Rheumatology.

[11] Li D., Wang H., He J.Y., Wang C.L., Feng W.J., Shen C., Zhu J.F., Wang D.L., Chen X.D. (2019). Inflammatory and fibrosis infiltration in synovium associated with the progression in developmental dysplasia of the hip. Mol. Med. Rep..

[12] Bastick A.N., Belo J.N., Runhaar J., Bierma-Zeinstra S.M. (2015). What are the prognostic factors for radiographic progression of knee osteoarthritis? A meta-analysis. Clin. Orthop. Relat. Res..

[13] Zhang Y., Nevitt M., Niu J., Lewis C., Torner J., Guermazi A., Roemer F., McCulloch C., Felson D.T. (2011). Fluctuation of knee pain and changes in bone marrow lesions, effusions, and synovitis on magnetic resonance imaging. Arthritis Rheum..

[14] Hill C.L., Hunter D.J., Niu J., Clancy M., Guermazi A., Genant H., Gale D., Grainger A., Conaghan P., Felson D.T. (2007). Synovitis detected on magnetic resonance imaging and its relation to pain and cartilage loss in knee osteoarthritis. Ann. Rheum. Dis..

[15] Perry T.A., Parkes M.J., Hodgson R.J., Felson D.T., Arden N.K., O’Neill T.W. (2020). Association between bone marrow lesions & synovitis and symptoms in symptomatic knee osteoarthritis. Osteoarthr. Cartil..

[16] Kraus V.B., Burnett B., Coindreau J., Cottrell S., Eyre D., Gendreau M., Gardiner J., Garnero P., Hardin J., Henrotin Y. (2011). Application of biomarkers in the development of drugs intended for the treatment of osteoarthritis. Osteoarthr. Cartil..

[17] Baker K., Grainger A., Niu J., Clancy M., Guermazi A., Crema M., Hughes L., Buckwalter J., Wooley A., Nevitt M. (2010). Relation of synovitis to knee pain using contrast-enhanced MRIs. Ann. Rheum. Dis..

[18] Loeuille D., Sauliere N., Champigneulle J., Rat A.C., Blum A., Chary-Valckenaere I. (2011). Comparing non-enhanced and enhanced sequences in the assessment of effusion and synovitis in knee OA: Associations with clinical, macroscopic and microscopic features. Osteoarthr. Cartil..

[19] Wallace G., Cro S., Dore C., King L., Kluzek S., Price A., Roemer F., Guermazi A., Keen R., Arden N. (2017). Associations between clinical evidence of inflammation and synovitis in symptomatic knee osteoarthritis: A cross-sectional substudy. Arthritis Care Res..

[20] Perry T.A., Yang X., van Santen J., Arden N.K., Kluzek S. (2020). Quantitative and semi-quantitative assessment of synovitis on MRI and the relationship with symptoms in symptomatic knee osteoarthritis. Rheumatology.

[21] Kaukinen P., Podlipska J., Guermazi A., Niinimaki J., Lehenkari P., Roemer F.W., Nieminen M.T., Koski J.M., Arokoski J.P., Saarakkala S. (2016). Associations between MRI-defined structural pathology and generalized and localized knee pain—The Oulu Knee Osteoarthritis study. Osteoarthr. Cartil..

[22] Wang X., Jin X., Han W., Cao Y., Halliday A., Blizzard L., Pan F., Antony B., Cicuttini F., Jones G. (2016). Cross-sectional and longitudinal associations between knee joint effusion synovitis and knee pain in older adults. J. Rheumatol..

[23] Felson D.T., Niu J., Neogi T., Goggins J., Nevitt M.C., Roemer F., Torner J., Lewis C.E., Guermazi A. (2016). Synovitis and the risk of knee osteoarthritis: The MOST Study. Osteoarthr. Cartil..

[24] Patry R. (1926). Contribution a l’étude des lesions traumatiques des menisques. La ménisco-synovite traumatique. Congrés Français Chir..

[25] Ayral X., Pickering E.H., Woodworth T.G., Mackillop N., Dougados M. (2005). Synovitis: A potential predictive factor of structural progression of medial tibiofemoral knee osteoarthritis—Results of a 1 year longitudinal arthroscopic study in 422 patients. Osteoarthr. Cartil..

[26] Shakoor D., Demehri S., Roemer F.W., Loeuille D., Felson D.T., Guermazi A. (2020). Are contrast-enhanced and non-contrast MRI findings reflecting synovial inflammation in knee osteoarthritis: A meta-analysis of observational studies. Osteoarthr. Cartil..

[27] Labinsky H., Panipinto P.M., Ly K.A., Khuat D.K., Madarampalli B., Mahajan V., Clabeaux J., MacDonald K., Verdin P.J., Buckner J.H. (2020). Multiparameter analysis identifies heterogeneity in knee osteoarthritis synovial responses. Arthritis Rheumatol..

[28] Siebuhr A.S., Petersen K.K., Arendt-Nielsen L., Egsgaard L.L., Eskehave T., Christiansen C., Simonsen O., Hoeck H.C., Karsdal M.A., Bay-Jensen A.C. (2014). Identification and characterisation of osteoarthritis patients with inflammation derived tissue turnover. Osteoarthr. Cartil..

[29] Petersen K.K., Siebuhr A.S., Graven-Nielsen T., Simonsen O., Boesen M., Gudbergsen H., Karsdal M., Bay-Jensen A.C., Arendt-Nielsen L. (2016). Sensitization and serological biomarkers in knee osteoarthritis patients with different degrees of synovitis. Clin. J. Pain.

[30] Kluzek S., Bay-Jensen A.C., Judge A., Karsdal M.A., Shorthose M., Spector T., Hart D., Newton J.L., Arden N.K. (2015). Serum cartilage oligomeric matrix protein and development of radiographic and painful knee osteoarthritis. A community-based cohort of middle-aged women. Biomarkers.

[31] Vilím V., Vytásek R., Olejárová M., Machácek S., Gatterová J., Procházka B., Kraus V.B., Pavelka K. (2001). Serum cartilage oligomeric matrix protein reflects the presence of clinically diagnosed synovitis in patients with knee osteoarthritis. Osteoarthr. Cartil..

[32] Saberi Hosnijeh F., Siebuhr A.S., Uitterlinden A.G., Oei E.H., Hofman A., Karsdal M.A., Bierma-Zeinstra S.M., Bay-Jensen A.C., van Meurs J.B. (2016). Association between biomarkers of tissue inflammation and progression of osteoarthritis: Evidence from the Rotterdam study cohort. Arthritis Res..

[33] Loeser R.F., Beavers D.P., Bay-Jensen A.C., Karsdal M.A., Nicklas B.J., Guermazi A., Hunter D.J., Messier S.P. (2017). Effects of dietary weight loss with and without exercise on interstitial matrix turnover and tissue inflammation biomarkers in adults with knee osteoarthritis: The Intensive Diet and Exercise for Arthritis trial (IDEA). Osteoarthr. Cartil..

[34] Arden N.K., Cro S., Sheard S., Dore C.J., Bara A., Tebbs S.A., Hunter D.J., James S., Cooper C., O’Neill T.W. (2016). The effect of vitamin D supplementation on knee osteoarthritis, the VIDEO study: A randomised controlled trial. Osteoarthr. Cartil..

[35] Roemer F.W., Kassim Javaid M., Guermazi A., Thomas M., Kiran A., Keen R., King L., Arden N.K. (2010). Anatomical distribution of synovitis in knee osteoarthritis and its association with joint effusion assessed on non-enhanced and contrast-enhanced MRI. Osteoarthr. Cartil..

[36] Bay-Jensen A.C., Wichuk S., Byrjalsen I., Leeming D.J., Morency N., Christiansen C., Karsdal M.A., Maksymowych W.P. (2013). Circulating protein fragments of cartilage and connective tissue degradation are diagnostic and prognostic markers of rheumatoid arthritis and ankylosing spondylitis. PLoS ONE.

[37] Perry T.A., Parkes M.J., Hodgson R., Felson D.T., O’Neill T.W., Arden N.K. (2019). Effect of vitamin D supplementation on synovial tissue volume and subchondral bone marrow lesion volume in symptomatic knee osteoarthritis. BMC Musculoskelet. Disord..

[38] Roemer F.W., Felson D.T., Yang T., Niu J., Crema M.D., Englund M., Nevitt M.C., Zhang Y., Lynch J.A., El Khoury G.Y. (2013). The association between meniscal damage of the posterior horns and localized posterior synovitis detected on T1-weighted contrast-enhanced MRI--the MOST study. Semin. Arthritis Rheum..

[39] Ballegaard C., Riis R.G., Bliddal H., Christensen R., Henriksen M., Bartels E.M., Lohmander L.S., Hunter D.J., Bouert R., Boesen M. (2014). Knee pain and inflammation in the infrapatellar fat pad estimated by conventional and dynamic contrast-enhanced magnetic resonance imaging in obese patients with osteoarthritis: A cross-sectional study. Osteoarthr. Cartil..

[40] Guermazi A., Roemer F.W., Hayashi D., Crema M.D., Niu J., Zhang Y., Marra M.D., Katur A., Lynch J.A., El-Khoury G.Y. (2011). Assessment of synovitis with contrast-enhanced MRI using a whole-joint semiquantitative scoring system in people with, or at high risk of, knee osteoarthritis: The MOST study. Ann. Rheum. Dis..

[41] Arendt-Nielsen L., Eskehave T.N., Egsgaard L.L., Petersen K.K., Graven-Nielsen T., Hoeck H.C., Simonsen O., Siebuhr A.S., Karsdal M., Bay-Jensen A.C. (2014). Association between experimental pain biomarkers and serologic markers in patients with different degrees of painful knee osteoarthritis. Arthritis Rheumatol..

[42] Bay-Jensen A.C., Bihlet A., Byrjalsen I., Andersen J.R., Riis B.J., Christiansen C., Michaelis M., Guehring H., Ladel C., Karsdal M.A. (2021). Serum C-reactive protein metabolite (CRPM) is associated with incidence of contralateral knee osteoarthritis. Sci. Rep..

[43] Organ L.A., Duggan A.R., Oballa E., Taggart S.C., Simpson J.K., Kang’ombe A.R., Braybrooke R., Molyneaux P.L., North B., Karkera Y. (2019). Biomarkers of collagen synthesis predict progression in the PROFILE idiopathic pulmonary fibrosis cohort. Respir. Res..

[44] Fukui N., Ikeda Y., Ohnuki T., Tanaka N., Hikita A., Mitomi H., Mori T., Juji T., Katsuragawa Y., Yamamoto S. (2008). Regional differences in chondrocyte metabolism in osteoarthritis: A detailed analysis by laser capture microdissection. Arthritis Rheum..

